# Diabetic Nephropathy: Significance of Determining Oxidative Stress and Opportunities for Antioxidant Therapies

**DOI:** 10.3390/ijms241512378

**Published:** 2023-08-03

**Authors:** Marina Darenskaya, Sergey Kolesnikov, Natalya Semenova, Lyubov Kolesnikova

**Affiliations:** Department of Personalized and Preventive Medicine, Scientific Centre for Family Health and Human Reproduction Problems, 664003 Irkutsk, Russia; sikolesnikov2012@gmail.com (S.K.); natkor_84@mail.ru (N.S.); kolesnikova20121@mail.ru (L.K.)

**Keywords:** diabetes mellitus, diabetic nephropathy, oxidative stress, biomarkers, antioxidant therapies, vitamins

## Abstract

Diabetes mellitus (DM) belongs to the category of socially significant diseases with epidemic rates of increases in prevalence. Diabetic nephropathy (DN) is a specific kind of kidney damage that occurs in 40% of patients with DM and is considered a serious complication of DM. Most modern methods for treatments aimed at slowing down the progression of DN have side effects and do not produce unambiguous positive results in the long term. This fact has encouraged researchers to search for additional or alternative treatment methods. Hyperglycemia has a negative effect on renal structures due to a number of factors, including the activation of the polyol and hexosamine glucose metabolism pathways, the activation of the renin–angiotensin–aldosterone and sympathetic nervous systems, the accumulation of advanced glycation end products and increases in the insulin resistance and endothelial dysfunction of tissues. The above mechanisms cause the development of oxidative stress (OS) reactions and mitochondrial dysfunction, which in turn contribute to the development and progression of DN. Modern antioxidant therapies for DN involve various phytochemicals (food antioxidants, resveratrol, curcumin, alpha-lipoic acid preparations, etc.), which are widely used not only for the treatment of diabetes but also other systemic diseases. It has also been suggested that therapeutic approaches that target the source of reactive oxygen species in DN may have certain advantages in terms of nephroprotection from OS. This review describes the significance of studies on OS biomarkers in the pathogenesis of DN and analyzes various approaches to reducing the intensity of OS in the prevention and treatment of DN.

## 1. Introduction

Diabetes mellitus (DM) belongs to the category of socially significant, non-infectious diseases with epidemic rates of prevalence. According to the International Diabetes Federation (IDF), 537 million people currently suffer from diabetes—a number that is expected to increase by more than 50% to 784 million by 2045 [1]. Disease prognosis is usually extremely unfavorable and, unfortunately, this trend has been maintained for many years [2]. Despite successes in studying the mechanisms of DM development and impressive results in the development of new drugs for the control of glycemia, the problems associated with DM are still increasing [3]. There are social and economic burdens that are determined by the development of micro- and macro-vascular complications, which are the causes of early disability and mortality of patients [4]. Clinical DM complications are mainly represented by stroke, coronary heart disease, peripheral artery disease, retinopathy, neuropathy and nephropathy [5].

## 2. Diabetic Nephropathy (DN): Pathogenesis and Existing Correction Methods

DN is a specific kind of kidney damage that occurs in 40% of patients with DM, according to recent data [6]. Chronic renal failure (CRF) due to DN significantly contributes to the mortality rate of patients with DM and is one of the main causes of mortality in patients with type 1 DM (T1DM) [7]. In patients with type 2 DM (T2DM), DN ranks third among the causes of death after cardiovascular diseases and oncological pathologies [8].

DN combines a number of changes in various renal structures (arteries, arterioles, kidney glomeruli and tubules) with the development of glomerular hypertension, often leading to the development of diffuse or nodular glomerulosclerosis and, subsequently, CRF [9]. A number of structural and functional events consistently occur in the kidney tissues of patients with DN. It is classically distinguished into several consequent stages: albuminuria, proteinuria with preserved kidney function and the progressive decrease in the functional activity of the kidneys until the terminal stage [10]. Currently, DN is not considered to be a fatal complication of DM since its development can be prevented.

Additionally, the possibility of the early preclinical diagnosis of DN has significantly expanded. Until recently, albuminuria was considered a key factor in glomerular damage (i.e., “albuminuria was a centric” model of pathogenesis) [9,11]. However, morphological studies have shown that the characteristic changes in kidney tissues are already present under the conditions of normal albumin excretion and that the detection of albuminuria indicates the presence of sclerosis in 20–25% of nephrons [12,13]. As a rule, by the time of the occurrence of persistent albuminuria (proteinuria), 50–70% of the renal mass has undergone sclerosis [13].

A key pathogenetic factor in DN development is persistent hyperglycemia [14]. Along with possible genetic predisposition (i.e., a smaller than usual number of nephrons in the kidneys, etc.), stable hyperglycemia is the basis for the formation of several complex and not yet fully understood pathological mechanisms that damage various renal structures (i.e., mesangial, tubular, interstitial and vascular structures) [10]. These effects are enhanced in the presence of various vascular risk factors (obesity, smoking, metabolic syndrome, etc.) [15]. Together, the impact of these factors is the formation of glomerular hyperfiltration (at the initial stage) and glomerular hypertension. Subsequently, the mechanism for autoregulating the renal tubular arteriole tone is disrupted due to systemic arterial pressure, directly affecting intraglomerular pressure, and glomerular hypertension develops from transient to constant [16]. The emergence of podocyte (glomerular epithelial cell) disorders and the development of podocytopathy are important factors in the development of albuminuria. Albuminuria, in turn, is considered an important mechanism for the further progression of glomerular damage, the formation of nodular glomerulosclerosis, the increase in the mesangial matrix, glomerular hyalinosis and tubulo-interstitial fibrosis [12,14]. At this stage, proteinuria and hypertension development is typical, which additionally stimulates renal structure damage processes and contributes to a decrease in kidney function, up to renal failure and the terminal stage [17].

Hyperglycemia has a negative effect on renal structures due to several factors, including the accumulation of advanced glycation end products (AGEs), the activation of the polyol and hexosamine glucose metabolism pathways, increased oxidative stress (OS) reactions, the activation of the renin–angiotensin–aldosterone system (RAAS) and the sympathetic nervous system and the increased insulin resistance and endothelial dysfunction of tissues [7,14].

It is unequivocally recognized that all the above mechanisms cause the development of OS reactions and mitochondrial dysfunction, which contribute to DN development and progression [18].

The main principle of DN prevention and treatment is the correction of metabolic and hemodynamic disorders, particularly the maintenance of good glycemic control (glycated hemoglobin (HbA1c) < 7%), the normalization of systemic blood pressure (<130/80 mm Hg), a reduction in intraglomerular hypertension and the elimination of dyslipidemia [9,10,13,14]. In this regard, HbA1c-lowering drugs, insulinotropic drugs, insulin-sensitizing drugs, anorectic drugs, incretin hormone mimic drugs, lipid-lowering drugs, drugs that prevent glucose reabsorption in the kidneys, etc., have become important [9,13,14,16]. RAAS blockers, such as angiotensin-converting enzyme (ACE) inhibitors and angiotensin II receptor blockers, remain the standard drugs approved for DN, according to international treatment algorithms [13,14]. Their nephroprotective properties have been evidenced in large clinical trials for the treatment of DN [9]. As a result, ACE inhibitors are recommended as the first-line treatment for DN in both types of diabetes [9,16]. At the same time, new classes of nephroprotective drugs are being developed that interrupt the chain of pathological changes in the kidneys caused by hyperglycemia or other factors (e.g., protein kinase C (PKC) inhibitors, cytokine and growth factor blockers, endothelin-1 antagonists, etc.) [9,13,14,16,17]. The idea of synthesizing drugs that could simultaneously exert hypoglycemic effects and nephroprotective effects that are not mediated by glycemic control is of great interest among scientists [16]. At the same time, most modern methods for treatment aimed at slowing down the progression of DN have side effects and do not produce unambiguous positive results in the long term [10]. As a result, alternative or additional approaches to DN treatment that produce maximum kidney protection are being studied [10,15,17]. These include various phytochemicals that have antioxidant activity as well as high activity against OS reactions, which are not only used for the treatment of DM but also other systemic diseases [3,6,9].

## 3. OS

During cell metabolism, free radicals (FRs) (otherwise known as reactive oxygen species (ROS) or reactive nitrogen species (RNS)) are continuously formed [19]. They can be of two types: some are the normal metabolic products of endothelial cells, which are caused by the release of super-oxides via phagocyte and nitric oxide (NO) activation, while others occur under altered environmental conditions (e.g., FRs in water and organic molecules that are synthesized under the action of ultraviolet, ionizing radiation, toxic substance or pathological conditions) [19,20,21]. FRs are highly reactive particles that contain unpaired electrons; that is, one or more electrons are missing in the outer orbital [22]. The main suppliers of FRs are mitochondria (mitochondrial nicotinamide adenine dinucleotide phosphate (NADPH) oxidase (NOX)) [23]. Superoxide radical (O_2_^•^) plays an initiating role in this process, since after formation in mitochondria, it can turn into more reactive forms, such as hydroxyl (^●^OH), hydroperoxyl and peroxyl (ROO^●^) radicals, hydrogen peroxide, singlet oxygen (^1^O_2_), nitric oxide radicals (NO^•^), nitrogen dioxide radicals (NO_2_^•^), etc. [19]. In low and moderate concentrations, ROS act as important elements in the cellular links of the immune system by regulating the synthesis of prostaglandins, leukotrienes and thromboxanes, as well as participating in the destruction of xenobiotic molecules, the renewal and modification of cellular biomembranes, the regulation of apoptosis, etc. [24]. ROS are regulators of the intracellular signal transmission pathways that control cell growth, differentiation and numerous other processes [18,22].

Under pathological conditions, the balance of ROS production and detoxification by the antioxidant defense system (AOD) is disturbed, which leads to system imbalance and OS development [20,21,22]. Sies provided the following definition of OS: “a violation of the balance between pro-oxidants and antioxidants towards the predominance of pro-oxidants, which leads to a violation of redox signaling and redox control and/or damage to molecules” [19]. The excessive production of ROS causes the oxidation of macromolecules (i.e., lipids, proteins and deoxyribonucleic acid (DNA)), which ultimately leads to modifications and changes that can persist for a long time [25]. OS increases vascular permeability, promotes leukocyte adhesion and causes changes both in the transduction of endothelial signals and the processes that are regulated by redox-sensitive transcription factors [26].

OS is currently considered an important pathogenetic link in various diseases, including cardiovascular diseases, oncological diseases, diabetes, arthritis, neurodegenerative disorders and lung, kidney and liver diseases [27,28,29,30]. The likelihood of developing these pathologies increases with age; therefore, OS is considered one of the main factors of aging and aging-related diseases [31]. Recently, a modern OS concept has been formulated, which asserts the necessary role of ROS in signaling, apoptosis and cell proliferation processes. This concept not only takes into account direct damage to ROS proteins, lipids and DNA, but also indirect effects via redox- or ROS-dependent signaling pathways. It also considers the relationship between OS and carbonyl stress, as well as distinguishing between systemic and local OS and considering the promising clinical assessment of pro-antioxidant status and the pathological correction of OS [19,24]. This concept could be applied to the development of diabetic angiopathies, including DN [32].

### OS Biomarkers

OS markers are important tools for assessing the status of redox potential and the course and progression of disease, as well as antioxidant effects [33]. ROS are extremely unstable and have short half-lives (only seconds), so it is almost impossible to measure them [24]. Unlike ROS, oxidation products from biomolecules last longer (from hours to weeks) and are commonly used to assess redox potential [22].

As a rule, the oxidation of macromolecules leads to the accumulation of certain products. For example, lipid peroxidation (LPO) produces malondialdehyde (MDA), thiobarbituric acid-reactive substances (TBARs), 4-hydroxynonenal (HNE) and F2-isoprostanes, while nucleic acid oxidation produces 8-hydroxyguanosine (8-OHG) and 8-hydroxy-2′-deoxyguanosine (8-OHdG), and protein oxidation produces advanced oxidation protein products (AOPPs), AGEs, methylglyoxal (MGO) and protein carbonyls [19,24,34]. The main way to protect against ROS is to neutralize them via the AOD system, which includes non-enzymatic antioxidants (i.e., reduced glutathione (GSH), vitamins (ascorbic acid, tocopherols, retinol, carotenoids, etc.), ceruloplasmin, ferritin, carnosine, etc.) and a wide range of antioxidant enzymes (i.e., superoxide dismutases (SODs), quinone oxidoreductase 1, catalases (CATs), glutathione peroxidases (GPx: eight isoforms) and peroxiredoxins (Prdx: six isoforms), glutathione S-transferases (GSTs), glutathione reductases (GRs), aldo-keto reductases, heme oxygenases, etc.) [35].

It should be noted that due to the ubiquitous and non-specific nature of OS, it makes sense to measure entire panels of biomarkers instead of just one since this reduces the likelihood of false positive and false negative results [36].

## 4. DN: The Significance of Determining OS

The kidneys are the second organ after the heart in terms of the number of mitochondria, which provide the best conditions for the synthesis of adenosine triphosphate (ATP) and the absorption of ultrafiltrate and dissolved substances [37]. The functions of renal structures depend on fatty acid β-oxidation and mitochondrial oxidative phosphorylation, during which a large number of ROS are formed [38]. Thus, the balance between the production of mitochondrial ROS and their neutralization via the AOD system is crucial for the normal function of mitochondria in the kidneys [39]. Most studies have claimed that mitochondrial dysfunction and the development of OS are the main pathogenetic factors responsible for the initiation and progression of DN [3,40]. At the same time, mitochondrial dysfunction is characteristic of various cells in renal structures, including endothelial cells and podocytes [41,42]. OS can mediate podocyte cell death, especially podocyte apoptosis, via many signaling pathways, as well as cell cycle arrest [43]. When certain pathways, such as *PI3K/Akt*, transforming growth factor β1 (TGF-β1)/p38-mitogen-activated protein kinases (*p38-MAPK*) and nuclear factor kappa B (*NF-kB*), are activated, this induces endothelial cell apoptosis, inflammation, autophagy and fibrosis, which cause histological and functional kidney disorders and, ultimately, kidney damage [44]. Recently, much attention has been paid to the iron-dependent process of LPO (ferroptosis), which has inspired a new idea for studying the progression of DN. Iron overload, reduced antioxidant capacity and massive amounts of ROS and LPO were all found in the kidneys of DBA/2J mice with streptozotocin (STZ)-induced diabetes, as well as in proximal tubule cells from human kidneys that were cultured with a high glucose content [45].

Hyperglycemia triggers a cascade of biochemical transformations, leading to damage to vascular walls and primarily activating the formation of super-toxic ROS molecules in mitochondria [14]. Inside the mitochondria, these reactive elements damage respiratory chain enzymes and mitochondrial DNA. Going beyond the mitochondria, reactive radicals trigger other mechanisms that are associated with the toxic effects of hyperglycemia, including vascular endothelial dysfunction, the formation of AGEs, the activation of PKC and *NF-kB*, epigenetic changes, etc. [46]. Despite that FRs live for less than a minute, proteins, fats and nucleic acids that have been damaged by them exist for a long time. AGEs also have a damaging effect on renal structures and their accumulation underlies the mechanism of “metabolic memory”, which is when modifications to biomolecules caused by ROS can lead to deviations in cellular function a considerable time after the initial manifestation of DM [47]. As a result, it becomes important to achieve good glycemic control within the first months of the occurrence of the disease. The spectrum of pathological effects of AGEs is extremely large. For example, they increase the synthesis of cytokines and growth factors, activate the processes of proliferation and sclerosis, activate platelet aggregation and thrombosis, affect gene expression and increase the frequency of mutations [36,47]. Most of all, vascular walls suffer from the effects of AGEs. The binding of AGEs to AGE receptors (RAGEs) stimulates the formation of ROS and leads to the development of OS [48]. AGEs and RAGEs are present in kidney cells and play significant roles in the induction of invasion, cell cycle arrest and proinflammatory changes in those cells [46]. AGEs can also increase the production of matrix metalloproteinases, which in turn cause kidney dysfunction and DN [44].

## 5. DN and OS: Experimental Studies

The administration of multiple consecutive injections of STZ is widely used to induce experimental models of DN as it selectively destroys beta cells in the pancreas, which leads to a decrease in insulin secretion [49]. In addition to its cytotoxic effect, STZ also contributes to the development of OS and mitochondrial dysfunction and the suppression of the activity of enzymes that destroy ROS. Thus, in rats with STZ-induced DM, changes in mitochondrial bioenergetics have been shown to precede the development of albuminuria and histological changes in the kidneys [50]. Mitochondrial fragmentation and decreases in ATP content have been observed in proximal tubule cells in the early stages (first four weeks) of DM in the absence of albuminuria and specific glomerular pathologies [51]. The progression of DM indicates mitochondrial dissociation, OS generation and glomerular changes [52]. The mitochondrial biosynthesis of ROS mediated by proliferator-activated receptor-γ coactivator 1-alfa (*PGC-1α*) coactivators and *NRF1* and *TFAM* transcription factors may be key in maintaining mitochondrial function [50,53]. Mitochondrial abnormalities, such as defective mitophagy, the formation of mitochondrial ROS and decreased mitochondrial membrane potential, have been reported in the glomeruli of db/db mice (which are used as models of obesity, DM and dyslipidemia), which were accompanied by a decrease in PINK expression and increased apoptosis [51,54].

In mouse models of diabetic kidney disease (DKD), it has been found that lactic acidosis and hypoxia contribute to mitochondrial abnormalities and fibrosis [55]. Rats with STZ-induced DM have been found to have high blood glucose and MDA levels, OS indices and general oxidant status [56]. High levels of inflammatory cytokines, the development of OS and nitrosative stress have also been noted [57,58]. MDA, NO, *NF-kB* p65 and tumor necrosis factor (TNF-α) levels have been shown to increase in the kidneys of rats with DN, while GSH, SOD and anti-apoptotic protein (Bcl-2) levels were reduced [59]. High glucose levels, decreased insulin, decreased kidney function, increased renal glomerular sclerosis and interstitial fibrosis, the high activity of OS reactions and the increased expression of TGF-β1, phosphorus-Smad2 and phosphorus-Smad3 have also been observed [60]. Significant decreases in the levels of GPx, GSH and SOD, as well as a significantly increased levels of MDA, have been reported in rats with DN [61], along with decreases in GSH, SOD and total antioxidant status (TAS) and increases in the levels of MDA and glucose in the blood of diabetic rats [62]. There have also been reports of increases in MDA and nitrite levels and myeloperoxidase activity and decreases in the activity of SOD, CAT and GSH [63]. Glomerular endothelial cells (GECs) become dysfunctional and pathological in relation to neighboring podocytes due to increases in the levels of mitochondrial super-oxides and increases in the frequency of DNA damage (8-OHdG) [42].

Endothelial NO synthases decrease, leading to the increased production of ROS and OS, which is associated with DN progression in experimental animal models [54]. In rats with DN, significant increases in glucose, blood urea nitrogen (BUN), N-acetyl-β-D-glucosaminidase and proteinuria levels have been found, along with concomitant decreases in the glomerular filtration rate (GFR), elevated levels of 8-OHdG, TGF-β1, MDA and GSH and reduced levels of GR and SOD [64]. Histological examinations have also revealed significant changes, including glomerulosclerosis and interstitial fibrosis, in diabetic groups [65]. Diabetic rats with STZ-induced DM have demonstrated hyperglycemia, which was closely associated with marked increases in MDA and protein carbonyl content, decreases in GSH, SOD and CAT levels in the kidneys and high levels of blood creatinine, BUN and urine albumin [66]. In addition, the increased expression of p65 *NF-kB* and proinflammatory cytokines (e.g., TNF-α, interleukin 1β (IL-1β) and IL-6) has been noted in the renal tissues of animal models [66]. Db/db mice have been shown to develop more kidney damage associated with molecular-1 and neutrophilic gelatinase lipocalin in the kidneys and urine, along with folds and disorders in renal tubule basement membranes, the accelerated formation of ROS, NOX and MDA and decreases in SOD, CAT and GPx levels [67]. The incubation of glyoxal (a byproduct of glucose autooxidation, involved in protein/lipid glycation and AGEs and LPO product formation) has been reported to reduce rat renal cell viability and lead to membrane lysis, the formation of ROS and LPO, mitochondrial membrane potential collapse and lysosomal membrane leakage [68]. AGEs have a toxic effect that contributes to the formation of DN via their accumulation in the vessels and various structures of the kidneys (i.e., mesangium, endothelium, podocytes, etc.). Experiments have shown that podocytes are the main target of AGEs, as evidenced by their expression of RAGE receptors [53]. Thus, in vitro, decreases in nephrin expression have been observed in podocyte cultures under the influence of glycated albumin, which shows its effects when combined with RAGE receptors. The damaging effect of AGE podocytes is also realized by activating apoptosis via the increased synthesis of the cell cycle inhibitor p27 [52].

## 6. DN and OS: Clinical Studies

In numerous clinical studies on DN development, the significant activation of OS reactions is in the form of increases in the serum levels of TBARs, MDA, AGEs, protein carbonyls and AOPP, as well as increases in urine 8-OHdG levels [40,69,70,71,72]. In patients with DM, a large number of oxidants are formed by non-functioning mitochondria and NOX1 in the liver [73].

A special pathogenetic role in DN is likely played by a set of negative factors that have damaging effects on renal tubules. Thus, it has been shown that increased OS, in combination with inflammation, cellular apoptosis and tissue fibrosis, leads to the steady progressive loss of kidney function and alters the glomerular filtration barrier [74]. The development of microalbuminuria is associated with insufficient glycemic control, hyperlipidemia, OS and the accumulation of AGEs [75]. In DN, the excessive accumulation of lipids in podocytes has been described, which leads to mitochondrial OS lipotoxicity, inflammatory reactions, actin cytoskeleton remodeling, insulin resistance (IR) and endoplasmic reticulum (ER) stress [53]. Hyperglycemia leads to the activation of signaling pathways and the production of ROS, as well as increases in the levels of cytokines and chemokines, such as IL-6, monocyte chemoattractant protein-1, TGF-β1 and vascular endothelial growth factor (VEGF), which consequently leads to inflammation, fibrosis and increased vascular permeability [76].

The hyperproduction of ROS leads to a consistent increase in TGF-β1, which contributes to the development of fibrosis in the tubulointerstitial parts of the kidneys [77]. In patients with DN, significant increases in glucose levels, carbonyl groups and ceruloplasmin, CAT and thiol levels have been reported [78]. Recently, the importance of the determination of LPO, VEGF primary products, podocyte damage markers and immuno-inflammatory factors in DN patients has been indicated [79]. According to our research, men with T1DM in the initial stages of DN demonstrate increased oxidative damage to lipids (primary products of LPO) and DNA. We also noted close relationships between these indicators and the duration of the disease [80,81]. It has also been noted that increases in the levels of circulating AGEs correlate with the increased risk of DN [79,82]. These authors found that synthesis growth in the initial stages of DN affected the RAAS and the functioning of TGF-β1, which contributed to chronic inflammation and glomerular and tubular hypertrophy [82,83,84]. It was also established that the activation of AGE-mediated receptors for AGEs (RAGEs) could cause the NOX-induced production of ROS and RNS, which subsequently led to OS and kidney aging in DN [83,84,85].

Elevated values of the carbonyl stress indicator MGO have also been observed in our studies [86]. 8-OHdG is a modified nucleoside base that is the product of oxidative DNA damage, which is formed by cutting oxidized guanosine from mitochondrial and nuclear DNA [19]. It has been found that the average level of 8-OHdG in the urine of patients with T1DM is significantly higher, especially in patients with persistent or intermittent microalbuminuria [87]. Data on the correlation between 8-OHdG and leukocyte content in urine and the severity of DN development are of particular interest, which confirms the importance of using these markers for predicting DN development and progression in patients with DM [88]. Increased values of this parameter have been observed in patients in the initial stages of DN, along with reduced values of telomere length [89]. In patients with DM and DN, increased levels of AOPP, a marker of protein damage, have been found to be more significant than in patients with DM without DN [90]. Decreases in the activity of the AOD system play a significant role in the pathogenesis of DN. Decreases in TAS, which reflects the overall activity of LPO inhibitors, undoubtedly have a negative impact on the AOD system state of patients with DN. Negative changes in TAS levels have been reported in patients in the initial stages of DN [91,92]. Decreases in vitamin E concentration are associated with the development of DM complications and play a significant role in the development of micro- and macro-albuminuria [3,92].

Statistically significant decreases in vitamin A, vitamin C and vitamin E concentrations with simultaneous increases in fasting blood sugar, postprandial blood sugar, blood urea, serum creatinine, HbA1c, sialic acid and microalbumin levels have been documented in the urine of patients with DN [93]. In patients with T2DM, DN and concomitant obesity, α-tocopherol and β-carotene levels in blood serum have been found to be below the optimal level, along with the significantly reduced urinary excretion of vitamins B1 and B2 and high vitamin D deficiency [94]. The same authors also found negative correlations between the ratio of vitamin C and E concentrations, glucosuria and postprandial glycemia, which indicated the need to maintain optimal levels of these vitamins [95]. Specific changes in the levels of tocopherol metabolites, including both short-chain and long-chain metabolites, have been reported, which regulate enzymatic and gene expression processes that are important for lipid metabolism and xenobiotic detoxification, as well as controlling immune and inflammatory processes [96,97].

In the literature, the available data on the activity of antioxidant enzymes in the initial stages of nephropathy are quite contradictory [3,97,98], which may be due to a number of factors, including the level of glycemic control, the duration of diabetes, concomitant complications, etc. [3]. Some researchers have noted that there are no differences in the activity of GR and GPx between patients with T1DM and controls [99]. However, other studies have recorded reduced GPx values [93]. The insufficient activity of this enzyme may indicate decreases in the utilization of phospholipids and hydroperoxides in fatty acids via the oxidation of GSH [42,100]. The loss of antioxidant genes, such as GPx4, and the activation of ACSL4 have also been observed in patients with DKD [53].

## 7. Opportunities for Antioxidant Therapies

The timely and dosed use of effective antioxidant agents is of particular importance in the treatment of diseases that have OS as their pathophysiological and pathobiochemical basis and is also necessary for the correction of pro-oxidant and antioxidant systems and oxidative metabolism in general. While in vitro studies have demonstrated the metabolic effects of antioxidant supplements, particularly with regard to the biology and activity of insulin [101], clinical studies are far from establishing the exact mechanisms of their action [97]. Modern antioxidant therapies for DN include various phytochemicals (food antioxidants, resveratrol, curcumin, preparations of α-LC, α-tocopherol, vitamin C, selenium, etc.), which are widely used not only for the treatment of DM but also other systemic diseases [102]. It has also been suggested that therapeutic approaches that target the exact source of renal ROS in DN may have certain advantages in terms of nephroprotection from OS [40].

### 7.1. Vitamins

The use of vitamins in patients with DN has shown promising results [102]. A large amount of data has indicated the potential roles of vitamins in slowing the progression of the disease and preventing the onset of its late stages [96].

#### 7.1.1. Vitamin E

Vitamin E is a fat-soluble vitamin that is an important micronutrient and a powerful antioxidant, which can prevent oxidative damage to the lipid components of cell membranes and participates in the modulation of protein and gene functions [103]. It exists in eight different forms: alpha (α), beta, gamma and delta tocopherols and alpha, beta, gamma and delta tocotrienols, with α-tocopherol being the most active form. Experimental and clinical trials have demonstrated the role played by vitamin E in preventing kidney damage [103,104]. Thus, α-tocopherol has the ability to modulate both tubulointerstitial damage and glomerulosclerosis, inhibit TGF-β1 expression and reduce MDA concentrations in plasma and the kidneys [104]. A 10-week intake of 600 IU of α-tocopherol has been shown to improve the levels of endothelial function biomarkers, the intracellular adhesion of molecule-1 and the vascular cellular adhesion of molecule-1; however, it had no effect on the serum concentrations of IL-6 or C-reactive protein (CRP) in patients undergoing hemodialysis [105]. The positive effect of tocotrienols and tocopherols on patients with DN with micro- or macro-albuminuria has been demonstrated [96]. In a randomized, double-blind, placebo-controlled study, it was demonstrated that taking vitamin E that was enriched with tocotrienol for 12 months could improve the progression of DN, especially in patients with stage 3 CKD (eGFR 30–60 mL/min/1.73 m^2^) [96]. Vitamin E supplementation that is enriched with tocotrienol has also been shown to improve kidney function, as evidenced by significant decreases in serum creatinine levels and significant increases in GFR levels compared to the controls [106].

It has been shown that tocotrienol helps to maintain glycemic levels in patients with DM and delay the appearance of diabetic complications due to its antioxidant, anti-inflammatory, antihypertensive and fibrolytic properties [107]. Vitamin E therapy for DN has been investigated using both oral vitamin E protocols and vitamin E-coated hemodialysis and has shown promising results in the secondary prevention of cardiovascular diseases, as well as immune and hematological complications [95,108]. Vitamin E intake can significantly reduce the levels of HbA1c, fasting glucose, fasting insulin and HOMA-IR in patients with DM, especially in patients with T2DM. The best dose of vitamin E for controlling HbA1c and insulin levels is from 400 to 700 mg/day; however, this prescription in patients with DN should be used with caution [109] as it is likely that vitamin E affects kidney function in various biochemical ways within the context of DN [96]. It has been found that the effectiveness of vitamin E for various cellular targets, such as podocytes, endothelial cells and mesangial cells [93], correlates with increases in the blood serum levels of proinflammatory mediators. Vitamin E supplements have been shown to reduce interstitial renal fibrosis and tubule epithelial cell apoptosis [96]. The results of one meta-analysis showed that short-term treatment with antioxidant vitamins could benefit patients with diabetes and albuminuria in terms of kidney function and systolic pressure; however, this treatment does not have a significant effect on glucose or lipid metabolism [92].

#### 7.1.2. Vitamin D

Vitamin D is involved in many physiological processes, and its active form (1,25(OH)2D3) modulates serum calcium–phosphate homeostasis and skeletal homeostasis [110]. Experimental studies, observational studies and clinical trials have shown the possible effects of vitamin D in terms of protection against DN progression and the preservation of the integrity of the glomerular filtration barrier; however, there are currently no recommendations for the optimal dosage or timeframe of vitamin D supplementation for the treatment of DN [110]. The use of vitamin D has been shown to significantly improve kidney podocyte function and reduce proteinuria [103]. A meta-analysis of 20 randomized clinical trials (RCTs) involving 1464 patients with DN showed that vitamin D supplementation had a beneficial effect on daily urine protein and inflammation indices in patients with DN, but not on serum creatinine, estimated GFR or glycemic control indices [111]. Vitamin D deficiency has been independently associated with microalbuminuria and reduced levels of high-density lipoproteins (HDLs) and positively associated with DN [112]. One study found that the prevalence of DN significantly increased with decreased serum 25(OH)D levels and that patients with DN had the lowest blood serum concentrations of 25(OH)D [113]. Vitamin D supplementation has been reported to significantly increase vitamin D levels and decrease serum levels of triglycerides (TGs), low-density lipoproteins (LDLs) and total cholesterol (TC) in patients with DN and T2DM compared to controls; however, changes in oxidative/antioxidant markers and HDL levels have been found to be insignificant after vitamin D intervention [114]. The selective activator of the vitamin D receptor paricalcitol has been observed to reduce proteinuria, slow kidney podocyte damage caused by high glucose levels via the regulation of key molecules (e.g., nephrin and podocin) and improve structural changes in mice with induced T1DM [115]. The use of calcitriol treatment in rats with DN has been found to reduce the severity of proteinuria and renal tubule epithelial cell apoptosis by activating vitamin D receptors, which inhibit p38-MAPK signaling [116]. Treatment with vitamin D has also been found to result in significant increases in insulin concentrations in rats with diabetes, which in turn could reduce hyperglycemia [117]. In addition, the expression of fructose-6-phosphate aminotransferase, a key regulatory enzyme in the hexosamine pathway, has been shown to be significantly reduced after the administration of vitamin D. An analysis of 9 RCTs involving 1547 people showed that patients receiving vitamin D treatment demonstrated statistically significant decreases in their urine creatinine/albumin ratios and albumin excretion coefficient values [118].

#### 7.1.3. Vitamin C (Ascorbate)

Vitamin C (ascorbate) is a water-soluble vitamin with active antioxidant activity, which can also restore other antioxidants. Vitamin C is found in large quantities in citrus fruits, green vegetables, etc. [119]. Some in vivo studies have revealed the potential pro-oxidant effects of this vitamin [3,93]. It has been reported that in diabetic rats, vitamin C treatment can significantly reduce proteinuria, albuminuria and the number of apoptotic cells and stop glomerular and tubulointerstitial sclerosis and the accumulation of MDA [101]. It has also been noted that vitamin C treatment, together with captopril, sodium hydrosulfide (NaHS), metformin and spironolactone, produces significant improvements in OS markers and renal hemodynamics [62]. Vitamin C can also significantly reduce LPO products, increase the activity of AOD enzymes (SOD, CAT, GPx, etc.) and decrease albuminuria and the thickness of glomerular basement membranes [119].

### 7.2. Resveratrol

Resveratrol (RESV) is a well-known natural phenolic compound that is found in plants, food and beverages and is the main component of traditional medicines, grapevines and red wine [6]. RESV has beneficial effects due to its cardioprotective, renoprotective, hepatoprotective, neuroprotective, anti-inflammatory, antidiabetic and antitumor properties [120]. It has been found that RESV can act as a good drug against DN since it actively participates in the regulation of various signaling pathways that are responsible for the production of ROS and AGEs, ER inflammation, the development of lipotoxicity, autophagy, the activation of the 5′adenosine monophosphate-activated protein kinase (*AMPK*) pathway and the modulation of angiogenesis [121]. It has been found that RESV protects against OS mainly by reducing the formation of ROS/RNS, the direct removal of FRs, the stimulation of the activity of endogenous antioxidant enzymes (for example, SOD, CAT, GPx, etc.), the stimulation of antioxidant molecules, the expression of related genes that are involved in the biogenesis of mitochondrial energy (mainly via the *AMPK/SIRT1/Nrf2, ERK/p38-MAPK and PTEN/Akt* signaling pathways) and the induction of autophagy via mTOR- or TFEB-dependent pathways [122]. The ability of RESV to stimulate AOD in DN has been demonstrated in a number of studies on cell cultures and preclinical trials [123,124]. In a diabetic rat model, it was found that RESV treatment had a beneficial effect on the mechanisms of “metabolic memory”, reduced quantitative BUN, serum creatinine and 24 h urinary microalbumin levels, improved the expression of inflammatory factors (RAGE, *NF-kB* (P65) and NOX4) and reduced pathological phenomena in the structure of the kidneys [125].

RESV treatment has also been found to prevent increases in MDA levels, the release of lactate dehydrogenase and the deposition of laminin and type IV collagen in diabetic kidneys due to its antioxidant effect and its modulation of the *Keap1/Nrf2* signaling pathway [126]. The effect of RESV has been shown to be tissue-specific: it reduces LPO in skeletal muscles, decreases GPx activity in blood serum and decreases CAT levels in the livers of Goto-Kakizaki (GK) rats (which were used as models of type 2 diabetes without obesity) [127]. RESV has been shown to effectively attenuate kidney damage by suppressing OS-mediated podocyte apoptosis, which depends on *AMPK* activation [128]. That study also revealed a possible mechanism for protecting podocytes from apoptosis in DN. A study on co-treatment with RESV (15 mg/kg/day) and ramipril (10 mg/kg/day) in rats with STZ-induced DN showed that glomerulosclerosis in the early stages of DN was reversible [129]. It has been found that RESV facilitates proteinuria, reduces the content of MDA, while increasing the activity of Mn-SOD in the renal cortex, inhibits the apoptosis of renal tubule glomerular podocytes and epithelial cells, improves pathological manifestations and restores the expression of the silencing information modulator 2 (sir2)-related enzyme 1 (*SIRT1*) and *PGC-1α*) in the kidney tissues of mice with DN [41]. In addition, these authors established its inhibitory effect on the production of mitochondrial ROS and its beneficial effects on the activity of complexes I and III in the respiratory chain, increases in mitochondrial membrane potential and the inhibition of the release of cytochrome C and DIABLO proteins from mitochondria into cytoplasm [41]. Their results showed that OS was the pharmacological target of RESV and that its powerful antioxidant properties probably significantly contributed to its protective effect against podocyte damage in mice with DN, which was mediated through the *SIRT1-PGC-1α* pathway [41,96]. Patients with DN demonstrate significant increases in AGEs, resulting from the binding of glucose to amino groups of proteins, lipids and/or nucleic acids in the bloodstream. These products have a significant damaging effect on kidney tissues [52,53].

RESV, curcumin and gallic acid inhibit the formation of ROS and LPO and weaken renal cytotoxicity caused by the action of glyoxal [68]. The administration of RESV (5 mg/kg during the last 45 days of a 90-day hyperglycemic period) to male Wistar rats with STZ-induced T1DM reduced renal hypertrophy, structural changes (i.e., tubular atrophy, mesangial expansion or wrinkling, diffuse glomerulonephritis and fibrosis), the accumulation of AGEs, OS, DNA damage (8-OHdG) and 4-HNE, caspase-3 and cleaved caspase-3 levels, but not RAGE expression [130]. RESV has an antioxidant effect in DN by modulating *Nrf2–Keap1* and related elements of the antioxidant response (ARE) and improving kidney function [131]. The oral administration of RESV (5 mg/(kg/day)) significantly improved kidney function, reduced the expression of proinflammatory cytokines and enhanced AOD by normalizing the expression of *Nrf2–Keap1* and its underlying regulatory proteins in rats with T2DM [121]. In addition, RESV suppresses ROS production in mesangial cells by suppressing the activity of NOX, which is a pro-oxidant enzyme and a generator of superoxide anions [6]. Combined therapy with RESV (20 mg/(kg/day)) and insulin can lead to maximum improvements in DN due to the hypoglycemic effect of insulin and the ability of both drugs to increase the activity of antioxidant enzymes in the renal cortex, inhibit LPO and activate Na+/K+−ATPase, independently of each other [132]. Moreover, the use of RESV as an adjuvant to traditional antidiabetic therapy may be an effective approach for the treatment of DN in humans. A study of 60 DN patients using losartan (12.5 mg/day) plus RESV (500 mg/day) for 90 days showed that RESV could be used as a potential supplement to angiotensin receptor blockers to reduce urinary albumin excretion in patients with DN [133].

Based on the above results, it may be concluded that RESV inhibits actions that are caused by hyperglycemic ROS formation and associated OS via multiple pathways, which has a pronounced nephroprotective effect on DN.

### 7.3. Curcumin

Curcumin (1,6-heptadiene-3,5-dione-1,7-bis(4-hydroxy-3-methoxyphenyl)-(1E,6E)) is a polyphenol compound derived from the traditional Chinese herb turmeric, which is commonly used as a spice and additive [134]. Numerous studies have shown that curcumin has positive biological properties, such as anticancer, anti-inflammatory, hypoglycemic, antioxidant and anti-apoptotic properties [135]. Based on the remarkable effectiveness of curcumin, scientists have begun to actively use it to treat DM and related chronic complications [136]. Curcumin can help to improve IR and glycemia control and reduce TG and TC in patients with T2DM [137].

When studying the effects of curcumin and metformin on OS in the kidneys of rats with T1DM, it was found that curcumin had a more pronounced effect and reduced MDA in kidney tissues, as well as restoring the overall antioxidant status and SOD, GPx and CAT activity [136]. In rats with STZ-induced DM, curcumin has been found to prevent renal tubule and mitochondria damage, mesangial cell expansion and basement membrane thickness. Curcumin also reduced the levels of inflammatory cytokines, improved the markers of mitochondrial function and suppressed the release of cytochrome C and the activation of caspase-3 [138]. The same authors found that curcumin lowered the levels of ROS, increased the levels of mRNA, MnSOD and gamma-glutamilligase and increased the levels of GSH and Bcl-2. Curcumin also prevented kidney damage in diabetic rats by activating *Nrf2*, inhibiting *NF-kB*, suppressing NOX and suppressing/inhibiting the *PKCßII/p66Shc* axis [138]. The antioxidant effect of curcumin is achieved by breaking the bond between *NrF2* and *KEAP1*, which triggers the activation of the *NRF2/KEAP1/ARE* pathway and leads to antioxidant enzyme synthesis [139,140]. Curcumin with chitosan nanoparticles improves podocyte activity in DN by reducing OS (decreased MDA and ROS levels with increased SOD activity) and inflammation (decreased TGF-β1, TNF-α and IL-6 levels with the increased secretion of IL-10) [141].

The administration of curcumin to diabetic rats with ischemia and reperfusion showed increases in inulin clearance, decreases in serum creatinine and NGAL and improvements in renal hemodynamics [142]. These effects were accompanied by decreases in oxidative and nitrosative metabolites and increases in thiol antioxidant reserves. Despite the wide spectrum of curcumin’s actions, it is characterized by poor solubility and poor absorption in its free form in the gastrointestinal tract, as well as rapid biotransformation into inactive metabolites [143]. Recently, nanocurcumin, which has better solubility, good digestibility and high efficiency, has been increasingly used in the treatment of DN [144,145]. In a randomized, double-blind, placebo-controlled study on nanocurcumin consumption in patients with DM on hemodialysis, significant decreases in fasting glucose, insulin, TG, very-low-density lipoproteins (VLDLs), TC, LDL, CRP and MDA levels were found, along with significant increases in TAS plasma content [146]. A qualitative analysis of 17 studies showed that polyphenolic intervention (including the use of curcumin) significantly reduced the levels of HbA1c, proteinuria and MDA, and increased GFR levels [147]. The daily oral administration of curcumin nanoparticles for 10 weeks, together with long-acting insulin, in rodent models of DN led to the significant suppression of renal NLRP3, IL-1β, *NF-ĸB*, Casp3 and *MAPK8* mRNA levels, which indicated the normalization of inflammation and apoptosis pathways [148]. In rats with STZ-induced DN, it was shown that the systematic consumption of curcumin (orally and through a gastric tube) weakened renal dysfunction, macrophage infiltration and fibrosis, probably due to its antioxidant properties [149].

### 7.4. α-Lipoic Acid

α-Lipoic acid (α-LA) (1,2-dithiolane-3-pentanoic acid or thioctic acid) is a natural dithiol that is enzymatically synthesized in plants, animals and humans and found in meat, the heart, kidneys and liver, fruits and vegetables [150]. α-LA is a powerful antioxidant due to its redox properties and its ability to directly capture ROS [151]. The common mechanisms of the protective action of α-LA for the kidneys, which include reducing oxidative damage, increasing antioxidant capacity, countering inflammation, softening renal fibrosis and weakening nephron cell death, have recently been identified [152]. α-LA also significantly reduces hyperglycemia, the loss of renal function, the expansion of the mesangial matrix and the development of glomerulosclerosis [40]. In diabetic rats, α-LA has been found to improve systemic glucose and urea levels, reduce AGE formation in the kidneys and maintain the structural integrity of the kidneys [153]. However, profibrotic phenomena provoked by DM were not facilitated by α-LA since collagen synthesis/deposition and the expression of TGF-β1 and α-actin in smooth muscles (α-SMA) remained elevated [153]. The α-LA supplement also partly restored the function of β-cells, promoted normoinsulinemia and normoglycemia, prevented renal pathology and significantly reduced elevated serum and tissue levels of MDA, collagen deposition, α-SMA expression, apoptosis and serum levels of IL-1β and IL-6. At the same time, α-LA significantly increased the content of GSH in the kidneys and the level of HDL in plasma [153]. α-LA probably prevents the progression of DN through the *p38-MAPK* pathway by reducing the expression of IL-6, TNF-α, *NF-kB* p65 and TGF-β1 [154]. Clinical trials on α-LA supplements have shown positive results regarding asymmetric decreases in dimethylarginine in DN patients [155]. α-LA has been reported to reduce the 24 h urinary albumin excretion and urine albumin/creatinine ratio of patients with DM, and the effect of α-LA alone did not differ from the results of studies in which α-LA was studied in combination with additional medications [156]. The use of α-LA in combination with valsartan significantly reduced urine levels of albumin and OS, increased the blood levels of SOD and TAS and facilitated renal dysfunction in patients with DN [157]. Our data showed decreases in the levels of TG, VLDL and Schiff bases and increases in the levels of retinol in patients with T1DM and DN [3,158]. The combined use of α-LA and α-tocopherol had a positive effect on kidney histopathology in a rat model with STZ-induced DM [159]. The administration of alpha lipoamide, a derivative of α-LA, significantly improved mitochondrial dysfunction and tubulointerstitial fibrous lesions in db/db mice, as well as increasing CDX2 and CFTR expression and decreasing β-catenin and snail expression in the kidneys of the mice [160]. 

### 7.5. Coenzyme Q10

Coenzyme Q10 (CoQ10), or ubiquinone, is a fat-soluble antioxidant, which is a key component of the electron transport chain in mitochondria, is necessary for the formation of ATP and actively participates in reducing the synthesis of ROS/RNS and cell biosubstrate modifications [161]. CoQ10 has shown promising clinical efficacy against DN as it affects OS reactions [147]. A meta-analysis of four RCTs and four experimental studies showed that the use of CoQ10 in combination with other drugs demonstrated a positive effect on the laboratory parameters of fasting plasma glucose, HbA1c, TC, HDL, TG and MDA levels [162]. Combined treatment with CoQ10 and N-acetylcysteine has been reported to improve STZ-induced renal damage (i.e., the normalization of histological characteristics, moderate changes in the appearance of glomeruli and tubules, decreases in OS intensity and increases in the activity of antioxidant enzymes) [63]. CoQ10, as an effective antioxidant in mitochondria, has also demonstrated a beneficial effect on DN in terms of mitophagy, restoring *Nrf2/ARE* signal transmission and reducing mitochondrial ROS levels [51]. Treatment with CoQ10 has been found to significantly reduce the levels of urea, creatinine and uric acid, as well as demonstrating clear improvements in renal tissues [163]. The synergistic effect of a 4-week treatment with lycopene (5 mg/kg orally) and CoQ10 (10 mg/kg orally) was manifested by decreases in the levels of biomarkers for renal disfunction and OS and increases in the levels of GSH and the activity of membrane-bound Na+/K+-ATPases [164]. CoQ10 intake by DM patients on hemodialysis for 12 weeks was associated with increases in TAS and NO levels and decreases in the levels of highly sensitive CRP, but it did not have any positive effects on MDA or GSH levels [165]. CoQ10 has been shown to normalize biochemical and antioxidant parameters, as well as histopathological characteristics, in experimental rats [166]. Combined CoQ10 and metformin therapy improved endothelial dysfunction and inflammatory changes in patients with T2DM, along with improvements in metabolic profiles [167]. Over the past few years, many analogs of CoQ10 have been developed to have improved characteristics, such as solubility in water and enhanced accumulation in mitochondria [168,169].

### 7.6. Other Methods of Protection against ROS

Over the past decade, mitochondrial-directed antioxidants (MTAs) have become a promising therapeutic option for the treatment of diseases caused by OS [170]. This has led to the development of a wide range of bioactive molecules and probes that can be delivered to mitochondria in vivo via oral, intravenous, intraperitoneal and subcutaneous administration [170].

Thus, the therapeutic efficacy of mitoquinone mesylate (MitoQ) was investigated in mice with DKD, and it was found that the intraperitoneal administration of MitoQ reversed tubule damage due to improved mtROS and mitochondrial fragmentation, which was caused by enhanced mitophagy mediated by *Nrf2* and PTEN-induced PINK1 kinase 1 [52].

Among the methods for protecting against excess ROS, a special place is occupied by the mimetics of antioxidant enzymes and substances that aim directly at the exact source of renal ROS in DN. It has been shown in various experimental models of kidney disease that catalytic antioxidants, especially the simulators of specific redox enzymes (such as SOD, CAT and GPx), have therapeutic benefits [171]. Treatment with recombinant human SOD1 significantly reduces ROS and improves kidney function by reducing TNF-α levels in tissues [172]. SOD1-transgenic mice have been found to show reduced albuminuria levels and the reduced expression of TGF-β1 and collagen IV, along with the expansion of the mesangial matrix and decreases in OS markers [173]. Pretreatment with recombinant SOD2 significantly increased SOD activity and improved renal function decline and tubular necrosis in a rat model of DN [174]. Therapy with ebselen, a GPx1 mimetic, has been found to have a protective effect on the development of atherosclerosis and DN due to decreases in proatherogenic biomarkers [175]. The inhibition of NOX activity by apocinin in mice with STZ-induced DM led to decreases in the signs of podocyte damage and albuminuria [176].

In a mouse model of DN with T1DM, the protective properties of SOD were demonstrated via decreases in ROS formation, the restoration of the expression of α3-integrins by podocytes and decreases in albuminuria [171]. The overexpression of CAT has been shown to weaken renal OS and prevent arterial hypertension, albuminuria, renal hypertrophy, tubulointerstitial fibrosis and apoptosis, as well as suppressing the expression of profibrotic and proapoptotic genes [170].

*NrF2* is a transcription factor and the main regulator of the AOD system, which comprises a set of detoxification genes that are activated in response to increased ROS levels [40]. *NrF2* increases the expression of various antioxidants, such as CAT, GSH, hemoxygenase-1, SOD and NADPH-quinone reductase [40]. Despite its increased expression in the initial stages of DN, the ability of this protein to translocate into the nucleus is reduced, which indicates an imbalance. A wide range of preclinical and clinical studies have shown the benefits of using *NrF2*-inducing strategies for the treatment of DN, such as the use of *NrF2* activators (e.g., bardoxolone methyl, curcumin, sulforaphane and their analogs) and other natural compounds with antioxidant properties [177,178]. The addition of *NrF2*-factor activators sulforaphane and cinnamic aldehyde in mice with STZ-induced DM led to the growth of detoxifying enzymes, the preservation of renal function and reductions in renal damage [179]. Epigallocatechin-3-gallate has the ability to indirectly increase *Nrf2*, mainly by activating the *Nrf2/ARE* signaling pathway at several stages, i.e., by suppressing *Keap1* and increasing the levels of *Nrf2* [179].

The inhibitors of NOX (which catalyzes the main pathways of ROS synthesis) are currently considered to be the most important components that suppress OS [40]. One study found that diosgenin inhibited the expression of NOX4, restored the expression of the I–V complex in the mitochondrial respiratory chain, reduced ROS production, facilitated pathological kidney damage and inhibited mitochondrial apoptosis, thereby improving the course of DN [180]. Thus, in a mouse model of DM, the administration of the inhibitor *GKT136901* reduced the production of hyperglycemia-induced ROS in proximal tubules and reduced the degree of albuminuria [178]. In addition, the administration of *GKT137831*, a low-molecular inhibitor of NOX1 and NOX4, to mice with DM revealed its renoprotective, anti-atherosclerotic and anti-inflammatory properties [128]. This substance also provides a high degree of renoprotection compared to that observed in NOX4-knockout mice [54]. A new pan-NOX inhibitor, APX-115, can prevent certain kinds of kidney damage, such as albuminuria, glomerular hypertrophy, tubule damage, podocyte damage, fibrosis, inflammation and OS [54].

The inhibitory effects of antidiabetic, antihypertensive and antidyslipidemic agents on OS have recently been proven [3,40,54]. Thus, in an animal model of DN, pioglitazone significantly reduced hyperglycemia, insulin resistance, glomerular sclerosis, hypertrophy, tubulointerstitial fibrosis and albuminuria, which contributed to decreases in OS [181,182]. It has also been established that the inhibitors of the sodium-glucose cotransporter 2 and the agonists of the glucagon-like peptide-1 receptor have beneficial effects on many pathophysiological disorders in DN, including OS and endothelial dysfunction [183].

## 8. Conclusions

T1DM and T2DM and their complications constitute serious public health problems worldwide and have high morbidity and mortality rates. DN is considered one of the serious complications and combines changes in various renal structures with the development of glomerular hypertension, often leading to the development of diffuse or nodular glomerulosclerosis and, subsequently, CRF. Most modern methods for treatments aimed at slowing down the progression of DN have side effects and do not produce unambiguous positive results in the long term. In most experimental and clinical studies, it has been claimed that OS and mitochondrial dysfunction are the main pathogenetic factors that are responsible for the initiation and progression of DN and that the balance between the production of mitochondrial ROS and their neutralization via the AOD system is crucial for the proper functioning of kidney mitochondria. Modern antioxidant therapies for DN include various substances (e.g., fat- and water-soluble vitamins, resveratrol, curcumin and α-lipoic acid preparations), which are widely used for the treatment of not only diabetes but also other systemic diseases. It has also been suggested that therapeutic approaches that target the source of ROS in DN may have certain advantages in terms of nephroprotection. Promising therapeutic options for the treatment of diseases caused by OS include mitochondrial-targeted antioxidants, which can be delivered to mitochondria in vivo via different administration methods. Thus, the measurement of OS biomarkers and AOD components may have advantages in terms of early diagnosis and intervention and deserves to be considered as an additional analysis tool. It is also important to develop individual approaches for treating patients with DN using therapeutic antioxidant agents.

## Data Availability

Not applicable.
